# Evaluating the landscape of social assessment methods: integrating the social dimension in sustainability assessment of product value chains

**DOI:** 10.1007/s11367-025-02432-z

**Published:** 2025-03-05

**Authors:** Nina van Dulmen, Carlos Felipe Blanco Rocha, Susana Toboso-Chavero, Reinout Heijungs, Jeroen Guinée

**Affiliations:** 1 Institute of Environmental Sciences (CML), Leiden University, Leiden, The Netherlands; 2 Department Circularity and Sustainability Impact, Netherlands Organization for Applied Scientific Research (TNO), Utrecht, the Netherlands; 3 Rotterdam School of Management, Erasmus University Rotterdam, Rotterdam, The Netherlands; 4 Integral Design and Management, Department of Materials, Mechanics, Management & Design, Faculty of Civil Engineering and Geosciences, Delft University of Technology, Delft, The Netherlands; 5 Department of Operations Analytics, Vrije Universiteit, Amsterdam, The Netherlands

**Keywords:** Social life cycle assessment, Reference scale approaches, Life cycle sustainability assessment, Safe and sustainable-by-design, Subcategory assessment method, Qualitative social assessment methods, Product value chains

## Abstract

**Purpose:**

We evaluate methodological approaches of different methods that can offer social assessments of product value chains. By analyzing both product-oriented social life cycle assessment (S-LCA) methods and qualitative, organization-, and project-oriented methods, we provide recommendations towards a clearer, harmonized method to better integrate the social dimension into sustainability assessments of products. This could help make S-LCA more analogous to environmental LCA (E-LCA) and more suitable for implementation in frameworks as life cycle sustainability assessment (LCSA).

**Methods:**

We apply two quantitative S-LCA methods side-by-side with three qualitative social assessment methods on the same case-study of a textile’s value chain. The two quantitative S-LCA methods adopt a quantitative functional unit (FU) approach, use similar data structures and calculation principles as E-LCA and are based on the product social impact life cycle assessment (PSILCA) database. The three qualitative methods applied include two social due diligence approaches — one based on the OECD Due Diligence and UN Guiding Principles for Business and Human Rights and the other on the IFC Performance Standards — and the Subcategory Assessment Method (SAM), a semi-quantitative performance evaluation assessment method based on the UNEP S-LCA Guidelines.

**Results:**

None of the approaches to S-LCA described in the UNEP S-LCA Guidelines can, at present, fully achieve the equivalent goals and scope of E-LCA, specifically in the social domain. Our evaluation of five social assessment methods, including two S-LCA methods, highlights their significant differences in basic structure and logic. Consequently, results differ considerably in nature, depth, and social aspects covered. Current product-oriented S-LCA approaches encounter important limitations as they require quantifiable aspects, whereas many social impacts are often qualitative in nature. Qualitative, organization-focused methods, conversely, make it difficult to link organizational social performance to specific products. Instead, these methods are typically used for social due diligence on suppliers in the company’s supply chain and cover only a small part of the product’s life cycle.

**Conclusion:**

For the purpose of computational integration, LCSA frameworks need an S-LCA method with a quantitative FU approach. However, only some S-LCA approaches are able to comply with this requirement, and these will only be able to cover a limited set of scalable quantitative impact indicators. We conclude by emphasizing the equal importance of product-oriented S-LCA and organization-oriented social assessment methods, while appreciating their fundamentally different goals and scopes.

## Introduction

Sustainability has a multidimensional nature, calling for the identification of environmental, economic, and social impacts across life cycle stages when assessing product sustainability. Life cycle thinking (LCT) offers a comprehensive approach to (re)consider product sustainability from a life cycle perspective (Arcese et al. 2017; Mazzi 2020), supported by life cycle sustainability assessment (LCSA). LCSA is an assessment framework including all three pillars of sustainable development (Klöpffer 2008), evaluating sustainability from three life cycle assessment methods: environmental life cycle assessment (E-LCA), life cycle costing (LCC), and social life cycle assessment (S-LCA) (Arcese et al. 2017; Klöpffer 2008; UNEP 2020). It is defined by Guinée et al. (2011) as a framework that broadens the scope of E-LCA to also include the economic and social pillars, broadening a purely product-level to include sector and economic levels of analysis, and going beyond only technical relations as physical or behavioral relations.

A comprehensive database of LCSA would include environmental, social, and economic data suitable for a sustainability-wide quantitative assessment of causal links between the unit processes triggered by product systems and their impacts (Heijungs 2010). Heijungs (2022) proposes a fully integrated computational structure of LCSA ($\mathbf{h}$ being the impact vector, $\mathbf{B}$ the satellite matrix, $\mathbf{A}$ the technology matrix, $\mathbf{f}$ the functional unit vector, and $\mathbf{Q}$ the characterization matrix) as the follows:

1 $${\boldsymbol{h}}=\left(\begin{array}{c}{{\boldsymbol{h}}}_{env}\\ {{\boldsymbol{h}}}_{econ}\\ {{\boldsymbol{h}}}_{soc}\end{array}\right)=\left(\begin{array}{ccc}{{\boldsymbol{Q}}}_{env}& 0& 0\\ 0& {{\boldsymbol{Q}}}_{econ}& 0\\ 0& 0& {{\boldsymbol{Q}}}_{soc}\end{array}\right)\left(\begin{array}{c}{{\boldsymbol{B}}}_{env}\\ {{\boldsymbol{B}}}_{econ}\\ {{\boldsymbol{B}}}_{soc}\end{array}\right){{\boldsymbol{A}}}^{-1}{\boldsymbol{f}}$$

In practice, however, S-LCA has been allowed to deviate substantially from the more similar E-LCA and LCC methods (Pollok et al. 2021). E-LCA — used to assess a product’s life cycle environmental impacts — evolved rapidly over the past decades as an acknowledged internationally standardized assessment method (Guinée et al. 2011) and is the most applied in LCSA (Mazzi 2020). LCC, in turn, assesses costs and benefits from economic activities related to a product system which can be integrated with E-LCA or used as a stand-alone method (Heijungs et al. 2012) and has an even longer history than E-LCA (Hunkeler et al. 2008). The social pillar is seen as “weaker” (Lehtonen 2004), regarding its level of methodological advancement of assessment methods and conceptual recognition (Valdivia et al. 2021). S-LCA faces barriers in terms of, e.g., (quantitative) data availability, leading to difficulties in adopting similar system boundaries to E-LCA, and shows great immaturity on methodological issues in impact assessment, results interpretation, and user-friendliness compared to LCC and E-LCA (Valdivia et al. 2021).

Compared to E-LCA or LCC, S-LCA is also more ambiguous, due to its more recent development and the current formulation in guiding documents on how to perform an S-LCA. Although early attempts to integrate social assessment into E-LCA exist (e.g., Dreyer et al. (2006); Norris (2006)), concrete development of S-LCA methods only began with the publication of the United Nations Environmental Programme (UNEP)/Society of Environmental Toxicology and Chemistry (SETAC) S-LCA Guidelines in 2009 (Ramos Huarachi et al. 2020). Since then, growing interest in life cycle sustainability assessment (LCSA) and S-LCA has been evident through published case studies and new methodological proposals (Guinée 2016; Ramos Huarachi et al. 2020). Additionally, upsurging frameworks such as “Safe and Sustainable-by-Design” (SSbD)^1^ (see, e.g., Caldeira et al. 2022; EC 2022) reflect the increasing use of LCSA, including S-LCA. Despite this growth, the current UNEP Guidelines for S-LCA (UNEP 2020) remain highly fragmented. For example, indicators can vary widely from qualitative to quantitative, and life cycle stage coverage can differ, resulting in a broad range of methods labeled as S-LCA. A wide variety of methods classified as S-LCA are developed and used by practitioners (Chhipi-Shrestha et al. 2015; Pollok et al. 2021). For this work, however, we define S-LCA as *solely those methods that use a quantitative, functional unit–based LCA structure and align with the computational framework of LCSA* outlined above.

This paper takes a broad look at how different approaches to social assessments of product value chains, including but not limited to S-LCA, can inform about the social sustainability of product value chains. We critically discuss the different characteristics, overlaps, strengths, and limitations of these methods while highlighting key elements that are needed for a clearer and more harmonized S-LCA method, that is feasible but also more compatible with integration in LCSA frameworks. We illustrate the different methods by applying them to a single illustrative case study of a textile value chain.

In Section 2, we explore key differences between social impact assessment methods. Section 3 briefly presents the case study, after which Section 4 presents the description and application of methods to the case study, assessing social risks of a T-shirt in a global value chain. Lastly, a discussion, conclusion, and outlook to the integration of the social pillar in LCSA and S-LCA’s compatibility herein can be found in Sections 5 and 6.

## Background

### S-LCA overview

The objective of S-LCA is evaluating social and socio-economic impacts and risks of products and services along their life cycle (Mazzi 2020; UNEP 2020). Several authors have worked on developing S-LCA methods in general (e.g., Dreyer et al. (2006); Hunkeler (2006); Norris (2006)). Currently, two main guidance documents exist: the UNEP Guidelines for S-LCA (UNEP 2020) and the Handbook for Product Social Impact Assessment — the latter based on the former (Goedkoop et al. 2020). The UNEP Guidelines (hereafter referred to as “the Guidelines”) are the most detailed and used guiding principles to S-LCA (Kühnen and Hahn 2017; Mesa Alvarez and Ligthart 2021) where databases like the Product Social Impact Life Cycle Assessment (PSILCA) database (Maister et al. 2020) or Social Hotspot Database (SHDB) (Bennema et al. 2022) are aligned to. Therefore, this key document is the basis of the evaluation of S-LCA in this paper.

S-LCA explores social impacts on people (i.e., stakeholders as potentially affected workers, local communities, or society), originating from the behavior of companies in a product’s life cycle^2^ towards these stakeholders (UNEP 2020). This perspective makes the relation of social impacts to a product system by nature different than with environmental impacts assessed through E-LCA, which instead primarily focuses on the physical aspects of unit processes (Dreyer et al. 2006). However, S-LCA still is being molded in an E-LCA-like framework; it adopts a quantitative E-LCA-structure involving unit processes and the formulation of a functional unit (FU). Quantifying this link between social impacts and a product system — i.e., scaling to a FU — is challenging and less straightforward than in E-LCA (Hauschild et al. 2008).

### Description and classification of existing approaches and methods

S-LCA employs two distinct impact assessment approaches: Reference Scale (RS S-LCIA) and Impact Pathway Life Cycle Impact Assessment (IP S-LCIA) (UNEP 2020). The Guidelines primarily outline guiding principles (not methods) — sometimes lacking clarity — focusing on characteristics of these two approaches.

One can use a fully qualitative to fully quantitative inventory for an S-LCA, depending on the type of impact assessment chosen (UNEP 2020), which is all still considered being S-LCA in the Guidelines. This, however, leads to having a fragmented variety of methods all being classified as S-LCA, that all have different scopes and retrieve various types of results. For example, the Guidelines (p57) specify that when one uses solely qualitative or semi-quantitative data, only the step of setting up a flow chart needs to be obtained in the inventory phase. The assessment then takes a further route evaluating qualitative data. When using quantitative data, additional steps need to be taken in the inventory phase; flow amounts should be obtained and scaled to the reference flow and eventually FU, and the assessment is done through common LCA software.

**Fig. 1:**
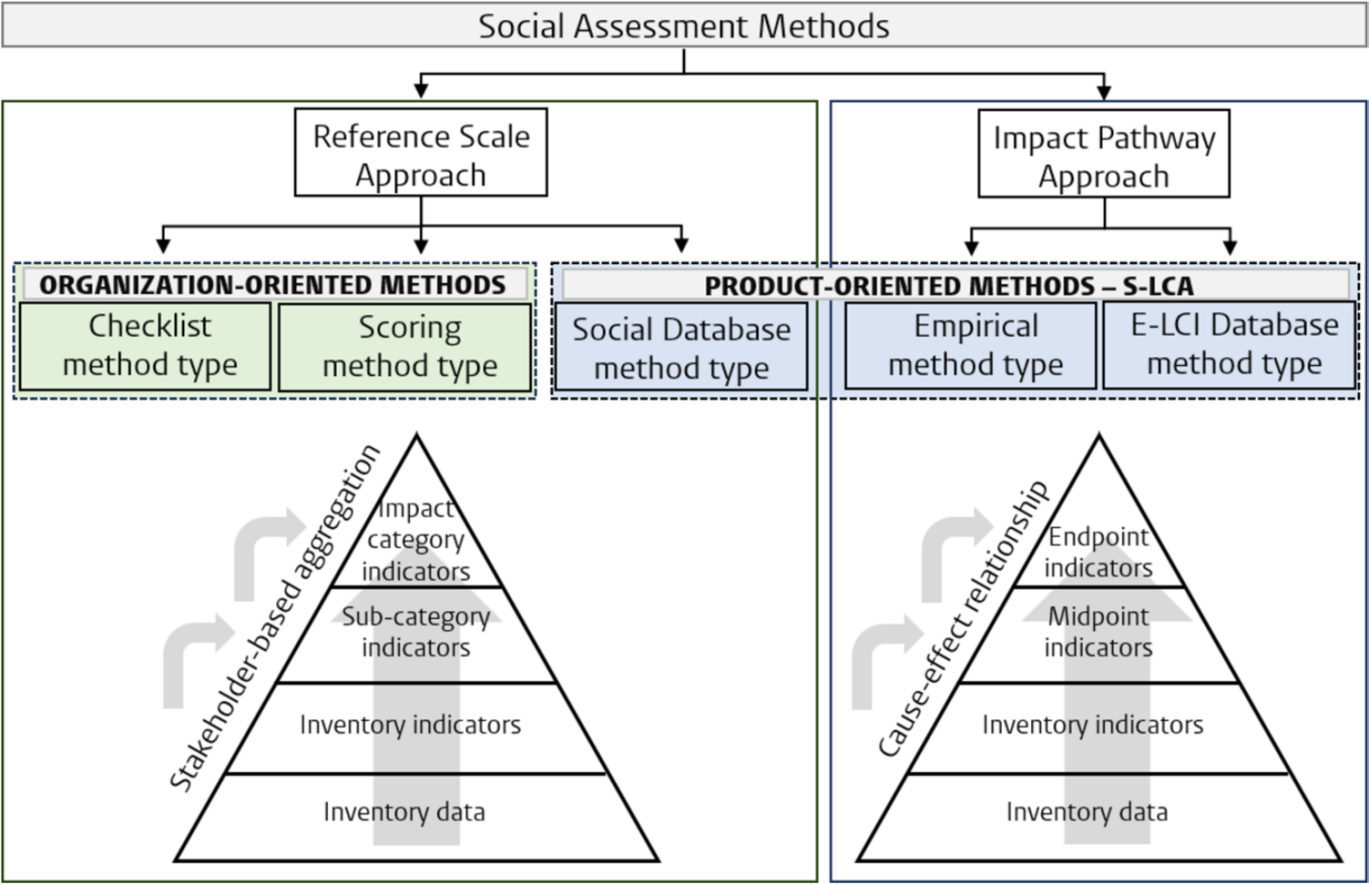
Social assessment approaches and their different methods and assessment levels. Adapted from Pollok et al. (2021) and Chhipi-Shrestha et al. (2015). Product-oriented methods (S-LCA; blue) are distinguished from organization-oriented (qualitative; green) methods

This ambiguity has results in a problematic variety in methods still being developed and used by practitioners (Chhipi-Shrestha et al. 2015; Pollok et al. 2021). Such methods (see, e.g., Harmens and Goedkoop 2021) are mostly developed based on the Guidelines and relying on the RS approach. Figure 1 presents different types of methods developed under RS and IP approaches. Through not solely taking a quantitative and product-oriented approach, not all RS methods fall under the term S-LCA as defined in this paper. The figure, therefore, distinguishes between *organization-oriented* and *product-oriented* methods: namely those that focus more on the behavior of value chain actors and those that adopt an E-LCA approach and scale social impacts to a FU (falling under S-LCA).

Reference Scale (RS) approaches utilize predefined reference scales, where organizational social performance or risks are assessed against. Such reference scales, exemplified in Table 1, can translate qualitative indicator-specific data (e.g., on child or forced labor or living conditions) to a semi-quantitative framework. For example, an organization is exhibiting “ideal performance” according to the left reference scale in Table 1 on the indicator “child labor” when it, e.g., has adequate policies, management systems, no child employment, and manages its tier-suppliers on this.

**Table 1:**
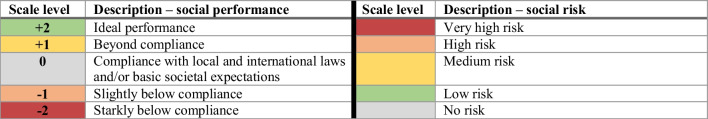
Exemplary generic reference scale for social performance (left) and social risk evaluation (right) (adapted from UNEP 2020). Reference scales may contain numbers specific to each level or just differentiate through colors

Qualitative RS approaches employ checklist or scoring methods to describe an organization’s social performance, whereas the quantitative RS “social database method” assesses social risks or potential impacts of products or services through using a social database (Fig. 1; Table 2) (Chhipi-Shrestha et al. 2015; Pollok et al. 2021). *Checklist methods* determine the presence or absence of a social issue, while *scoring methods* assign scores to assess these issues, introducing variability due to the lack of a standardized approach (Chhipi-Shrestha et al. 2015). These two methods do not allow for an aggregation over a product’s life cycle and do not take a quantitative approach, and thereby, there are no methods under S-LCA as described in this paper.

**Table 2:** Key characteristics of different RS and IP approaches

**RS approach**	**Key characteristics**
Checklist method type	• Organization-oriented method • Allows for assessing the presence or absence of an impact and/or checklist item • Assesses social performance of organizations, through, e.g., requirement compliance assessment
Scoring method type	• Organization-oriented method • Benefits from a before-hand screening on relevant social issues to assess for data collection feasibility reasons • Assesses social performance of organizations, through identifying the performance level by assigning scores (Chhipi-Shrestha et al. 2015) (most commonly based on an established set of reference scales)
Social database method type	• Product-oriented method and S-LCA • Uses generic country/sector-level data, readily available through social databases (PSILCA or SHDB) • Assesses product-level social risks, performance, or potential social impacts over a product’s life cycle through common LCA modeling with the ability of foreground modeling and including background processes through the use of the database. Scale impacts to a FU and is the RS-approach under S-LCA
**IP approach**	**Key characteristics**
Empirical method type	• Product-oriented method and S-LCA • Cause-effect modeling through using empirical relationship pathways (e.g., Preston or Wilkinson)
E-LCI database method type	• Product-oriented method and S-LCA • E-LCI database as basis for social assessment • Conducting E-LCA focusing on the more “social” indicators, e.g., focusing solely on human health impact categories

The *social database method* — the RS S-LCIA approach under S-LCA — involves utilizing databases as the widely used SHDB (Bennema et al. 2022) or PSILCA (Maister et al. 2020) (Mesa Alvarez and Ligthart 2021). These databases host data on social risks on country-, sector-, and stakeholder level (Pollok et al. 2021). Sectorial risks — or specific company behavior when primary data is available — are commonly linked to a product system through a so-called *activity variable* (typically *worker-hours*, or less-commonly used *value-added*) (Appendix I). This variable expresses the time spent on labor associated with a specific unit process to produce a certain output (UNEP 2020). Through this variable, social indicators are linked to the product system, allowing for life cycle aggregation. Nevertheless, individuals beyond the realm of workers, such as consumers or society, harbor distinct connections with products that extend beyond the hours devoted to labor. This correlation exclusively pertains to the stakeholder category “workers.” The activity variable — initially applied across all indicators — thus proves imperfect for other stakeholder categories: social factors lacking a direct causal relationship with labor time, like the unemployment rate in a country, become inaccurately linked to a product system through this variable. Also, using the variable causes communication issues associated with expressing results as, e.g., “med risk hours” (Ciroth and De Bellis 2020). Therefore, efforts are made to find alternatives to using such a variable, though still adopting a FU and allowing for life cycle calculations.

The Impact Pathway (IP) approach is still very underrepresented in S-LCA studies (Chhipi-Shrestha et al. 2015; Pollok et al. 2021). With IP S-LCIA, the magnitude of social consequences to a product system are assessed using mid- or endpoint indicators (UNEP 2020). Through purely quantitative cause-effect modelling, it portrays causal or correlation/regression-based relations as changes in the life cycle of a specific product, projected to the endpoint “human well-being” (simply “happiness”) (UNEP 2020). The Guidelines argue *potential social impacts* or *actual social impacts* can be calculated through IP S-LCIA. The latter are defined as stakeholder-affecting consequences from an activity’s *causal* relation to human well-being, based on observed and verified primary data (UNEP 2020). The nature of human interaction and uncertainty of behavioral impact (Pollok et al. 2021), as well as the lack of a time dimension in data, however, leads to few impacts being labeled as “actual.” Through its mid- and endpoint cause-effect modeling, IP S-LCIA methodologically comes closest to E-LCA as compared to RS S-LCIA.

Two methods fall under IP S-LCIA (Fig. 1; Table 2) (Chhipi-Shrestha et al. 2015). Through the *empirical method*, causal links are assessed by empirical relationship pathways as the Preston pathway (describing the relationship between economic activity and life expectancy) or Wilkinson pathway (describing the relationship between income inequality and health). Frequently, however, existing data does not allow for such quantitative cause-effect modeling as this relationship is often unknown (Pollok et al. 2021). Lastly, the *E-LCI database method* uses E-LCI (i.e., Life Cycle Inventory) databases for assessing social impacts. This, however, implies, e.g., conducting an E-LCA focusing solely on human health impact categories, using DALY as an indicator (Chhipi-Shrestha et al. 2015).

Acknowledging the more recent development of IP S-LCIA over RS S-LCIA (UNEP 2020), the Guidelines currently steer the reader towards the latter. As the Guidelines mention that within S-LCA different methods have different purposes, different paths are presented that one can take for this assessment (UNEP 2020). However, the Guidelines seem to focus on suggesting the utilization of a social database method and adopting a RS approach. Social databases are emphasized as primary sources of data, with a recommendation that these databases should complement on-site data collection to ensure the comprehensive capture of social impacts associated with the product system (UNEP 2020, p. 13). Additionally, the Guidelines highlight that many S-LCA studies utilizing activity variables achieve this through the use of S-LCA databases as PSILCA or SHDB (p65) (UNEP 2020). As far as the authors are aware, practical S-LCA methods developed under the IP approach are yet to be established, as evidenced by the absence of relevant case studies. The insufficiency of practical case studies, coupled with the fact that the Guidelines solely delineate the overarching principles of IP S-LCIA, hinders a comprehensive understanding of the operational mechanisms underlying these aforementioned methodologies. Consequently, evaluating this approach is considered beyond the scope of this research and case study.

## Illustrative case study of textiles

**Fig. 2:**
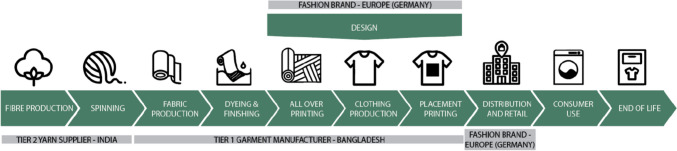
Value chain mapping of the three main tier suppliers. Own image. Tier 1 is a direct supplier of the product, whereas tier 2 suppliers are suppliers for the tier 1 supplier

As introduced above, our case study focuses on the textile value chain, and we define the FU for the illustrative S-LCA study as “Wearing an average weight T-shirt over a period of washes corresponding with typical use behavior in Germany.” This T-shirt, taken as an illustrative case, has a global value chain (Fig. 2): cotton is harvested and spun in India, after which T-shirts are manufactured in Bangladesh, which ultimately reach the European market. For S-LCA modeling purposes, Germany is modeled specifically, as this is the country where most sales take place. Three key organizations are involved: (i) the Tier 2 yarn supplier in India, (ii) the Tier 1 T-shirt manufacturer in Bangladesh, and (iii) the fashion brand located in Germany. Primary data is sourced from these three companies, as other Tier 2 organizations play a lesser role in material provision.^3^ In the S-LCA model, these additives — i.e., process chemicals — are modeled using sector-level data. Regarding the use phase, energy and water requirements are considered, and the end-of-life stage is not included due to data unavailability.

## Application of methods to case study

### Selection of methods

**Fig. 3:**
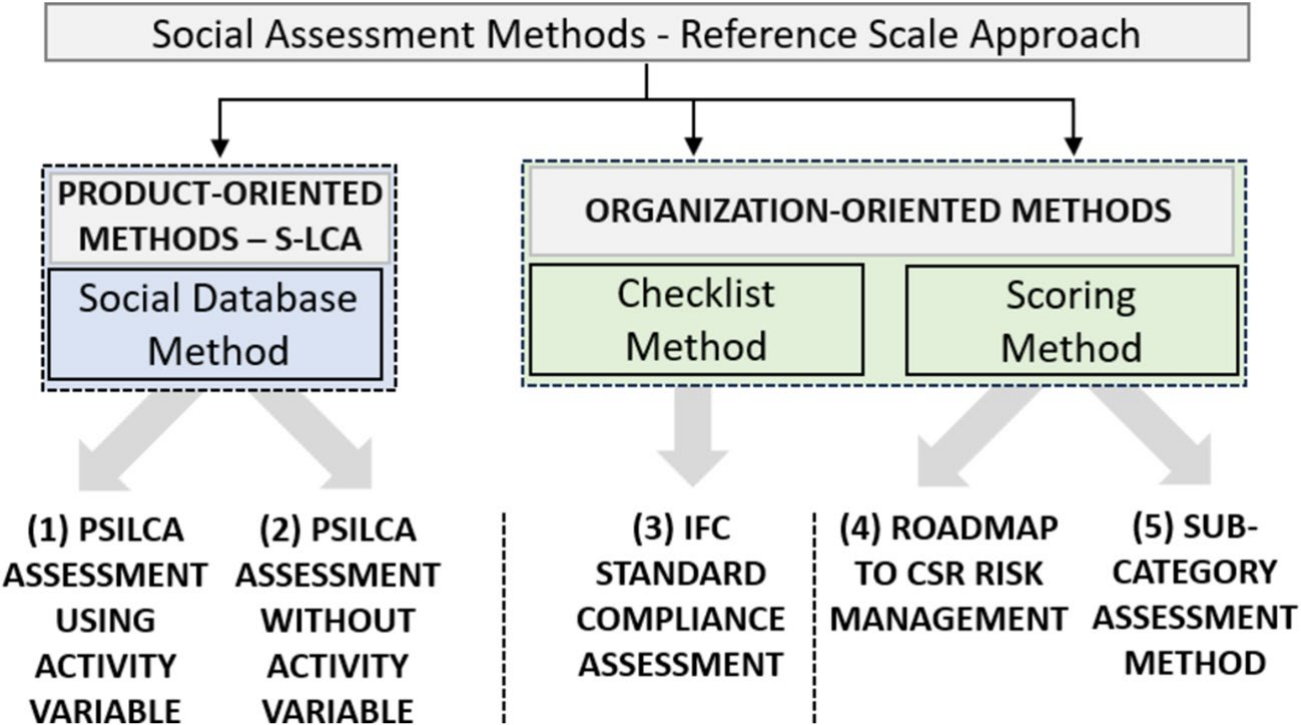
Five RS methods performed on the case study assessing the social impacts of a T-shirt with a global value chain

Five methods under the RS approach were applied to the case study (Fig. 3). The two S-LCA social database methods are conducted using the PSILCA social database (through datasets with and without using an activity variable). Furthermore, two qualitative and organization-oriented methods are performed, an International Finance Corporation (IFC) Performance Standard (IFC 2012) Compliance assessment and the Roadmap to CSR Risk Management (MVO 2020) (based on the Organization for Economic Co-operation and Development (OECD) due diligence and United Nations Guiding Principles (UNGP) for business and human rights). These methods take a social due diligence approach, are not connected to S-LCA, and we evaluate them as checklist and scoring method respectively. Lastly, the Subcategory Assessment Method (SAM) (Ramirez et al. 2014), is evaluated as an organization-oriented and semi-quantitative scoring method, initially developed as a more qualitative impact assessment method to S-LCA. However, through not scaling impacts to a FU in a quantitative manner, this approach is not considered S-LCA by our definition of S-LCA in this paper. A more detailed documentation of the application of these methods to the case study can be found in van Dulmen (2023). In the following sections, we summarize the key aspects.

### S-LCA Social database methods

#### PSILCA — Activity variable method

##### Description

The PSILCA database (outlined in Appendix I) includes a set of 69 indicators aligned with the 2009 UNEP/SETAC S-LCA Guidelines (Maister et al. 2020). Sector-level indicators^4^ or social flows (equivalent to environmental flows in E-LCA) are associated with indicator-level risks (Maister et al. 2020), categorized across six levels ranging from “no risk” to “very high risk.” Indicators are linked to a product system through the “worker-hours” activity variable as unit, representing the labor hours required to produce 1 USD of output — as the database is monetary-based. For example, social flows for the yarn production in India are sourced from PSILCA’s “Manufacturing of textile” sector, with the “Drinking water coverage” flow defaulted to “very high risk.” Consequently, this risk-assessed flow is allocated an hourly unit of — in our case — 0.25 h of labor per 1 USD output.

Equation 1 implies that for LCSA integration, only the satellite (B) matrix should vary among E-LCA, S-LCA, and LCC: assuming identical system boundaries represent a common A matrix. In practice, however, system boundaries do differ. Particularly in S-LCA, the fact that the PSILCA database is sector based — not activity based — limits using the database for modeling only available processes — often using proxies, compared to the broader inventory modeling for E-LCA through more extensive databases. Moreover, the distinction between S-LCI and S-LCIA is less clear than in E-LCA, as flows are already risk-assessed in the inventory phase. PSILCA’s “Social Impact Weighting Method” translates risk-assessed flows to a uniform medium-risk level across impact categories using arbitrary characterization factors. For instance, a low-risk level has a characterization factor of 0.1, whereby multiplying the low-risk worker-hours value by this factor yields its equivalent in “medium risk hours” (see Appendix I).

##### Case study application and illustrative results

For inventory modeling, we first mapped the product value chain and aligned this with sectors available in the PSILCA database to derive social flows. When primary data validated inapplicability of certain flows (e.g., no child labor at the manufacturing facility in Bangladesh), these simply were omitted. As direct monetary data was unavailable, our next step involved converting physical LCI data into monetary values using product values from ecoinvent and adjusting them to match PSILCA’s currency and year (USD2011) — via exchange rates, following the approach outlined by Koese et al. (2022) (see Appendix I). As a third step, for quantifying social flows for each unit process, default worker-hour values from PSILCA were used, in the absence of primary data.

Impact assessment results are expressed as “med risk hrs,” by multiplying the characterization factor of risk-assessed flows with the amount of worker-hours (selection of results presented in Table 3). The higher the med risk hrs value, the more risk is associated with the social flow. However, these values alone do not allow for any further interpretation.

**Table 3:**
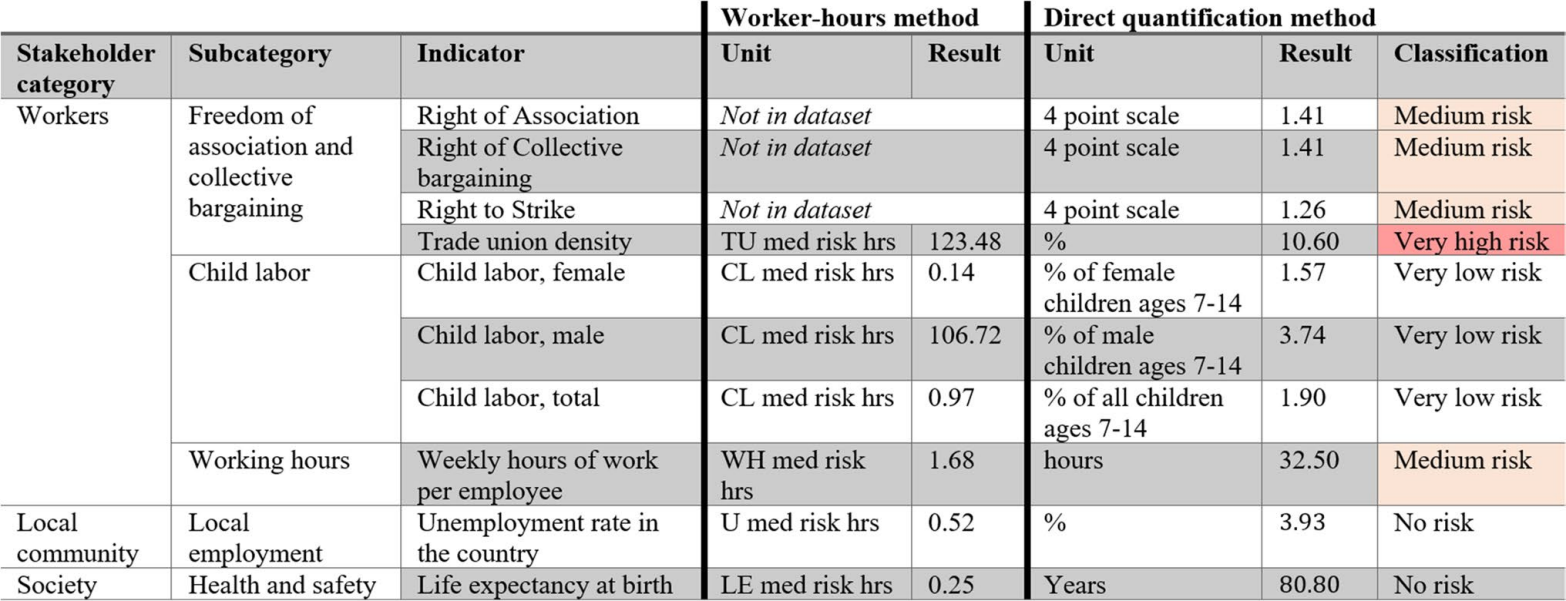
Selection of impact assessment results (case study) — PSILCA worker-hours and direct quantification methods

#### PSILCA — direct quantification method

##### Description

Alternative to the activity variable method, aiming at improving the interpretability of results, PSILCA developers propose a “direct quantification” method — which can be performed through a separate PSILCA version 3 dataset. This method calculates potential social impacts using non-risk-assessed inventory flows (Ciroth and De Bellis 2020), bypassing the need for an activity variable. By presenting “actual” indicator values (over presenting these as risk-classes), the method addresses communication issues associated with expressing impact assessment results as “medium-risk hours” (Ciroth and De Bellis 2020).

This method involves no characterization step, leaving calculations at inventory level. Having a totally different basic structure compared to the activity variable method, this particular method deviates even further from the E-LCA framework — and Eq. 1. Inventory social flows are directly related to their indicator with “raw”/non-risk assessed values, meaning they do not reflect a number of worker-hours or risk class. For example, child labor is expressed as the “% of all children ages 7–14” and life expectancy at birth is expressed in “years.” The method normalizes^5^ overall results by the total amount of products in a life cycle, assuming that each process contributes a proportionate share of its product to the overall results and consequently to the final impacts (Maister et al. 2020). Practical implementation of this concept involves dividing all results by the scaled diagonal of the A matrix (Maister et al. 2020). Hereby, the method does not allow for performing a contribution analysis. Manual risk categorization (i.e., assigning risk levels), however, can still be done using default risk-classes PSILCA database and assigning these to final inventory results, translating these average conditions to a semi-quantitative risk scale.

##### Case study application and illustrative results

Firstly, the product system is modeled similarly to the activity variable model, differing only in how social flows are quantified through their raw units. These flows are quantified using default values retrieved from relevant sectors in the database, such as a 1.7% value on child labor (of children ages 7–14), retrieved from PSILCA’s “Manufacturing of Textiles” sector in India. Lastly, where possible and when specific data was available, these values were adjusted to better reflect the specific primary process.

Since results represent averaged social conditions across the value chain (selection of results presented in Table 3), this method does not facilitate performing a contribution analysis. For example, results show a “very high risk” pertains to indigenous people’s protection. Yet, pinpointing the origin of this risk within the product system is not possible. A risk categorization step can manually be done (most right column of Table 3) through classifying results in risk-classes, following the default risk-classes of the database (Maister et al. 2020).

### Checklist methods

#### IFC Compliance assessment

##### Description

As a qualitative and organization-oriented checklist method, an IFC Compliance assessment was conducted. Performance Standard 2 (IFC 2012) presents a list of requirements on labor and working conditions. It covers aspects on safe and healthy working conditions, avoidance of forced and child labor, and identification of supply chain risks. Where IFC Performance Standards are not directly a social impact assessment method, we used the formulated requirements in Standard 2 as criteria to assess compliance of company-specific policies against.

##### Case study application and illustrative results

This IFC Standard Compliance assessment analyzes the company’s policy-adherence to IFC Standard 2 requirements (selection of results presented in Table 4). Policy documentation of involved organizations was gathered, for a policy-level compliance assessment onto human resource policies and procedures, health, and safety and working conditions. These policies were assessed against the Standard, simply on (i) full compliance, (ii) medium/partial compliance, (iii) no compliance, (iv) unknown, or (v) not-applicable. It was found that due to a lack of data, it was difficult to perform the assessment on the Tier 2 supplier. Of the three organizations, the fashion company adheres to most of the listed requirements.

**Table 4:**
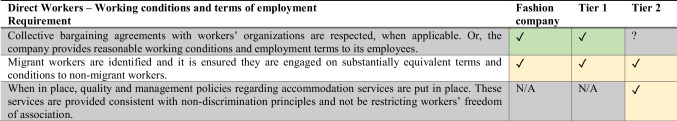
Selection of results of the IFC Compliance assessment on requirements of Performance Standard 2, specifically for direct worker’s conditions and employment term. Green = full compliance, yellow = medium compliance, and ? = unknown

### Scoring methods

#### Roadmap to CSR risk management

##### Description

The Roadmap to CSR Risk Management (MVO 2020) (hereafter “the Roadmap”) was identified as a more qualitative and organization-oriented due diligence method, guiding businesses with their CSR risk management. The Roadmap requires mapping of a specific supply chain, after which the associated CSR Risk Tool (MVO 2020) identifies sector-level risks related to this product system. This tool generates a detailed report on risks for specific sector/country level. Such reports can help investigate actual risks in the specific supply chain, through evaluating these sector-level risks to the supply chain, using organization-level characteristics found in, e.g., policy-level documentation. This leads to a list of potential risks for a specific product or service, which can be evaluated on their likelihood and severity for prioritizing risks to take action on (MVO 2020).

##### Case study application and illustrative results

We utilized the Roadmap as a framework to evaluate social performances of value-chain organizations. Firstly, we collected CSR policies and activities of the companies, through CSR Reports, policy documents, and interviews. Hereafter, sector-level risks were identified using reports created through the CSR Risk Check Tool (MVO 2020) on the yarn production in India and T-shirt manufacturing in Bangladesh. Other parts of the value chain were omitted due to either the sector, e.g., design and retailing, not being available in the tool or having insufficient primary information for sectors as, e.g., shipping from Bangladesh to Europe.

Sector-level risks through the generated report on garment manufacturing in Bangladesh were then qualitatively assessed against company traits. Based on the likelihood and severity of the specific risk, a rating was added to each. For example, there is a recognized sector-level risk of forced labor in Bangladesh. Yet, the Tier 1 manufacturer, with strong policies against forced labor, does not engage in such practices. Despite the risk’s severity, its likelihood at this company is low, resulting in a “Monitor” rating (Table 5).

**Table 5:**
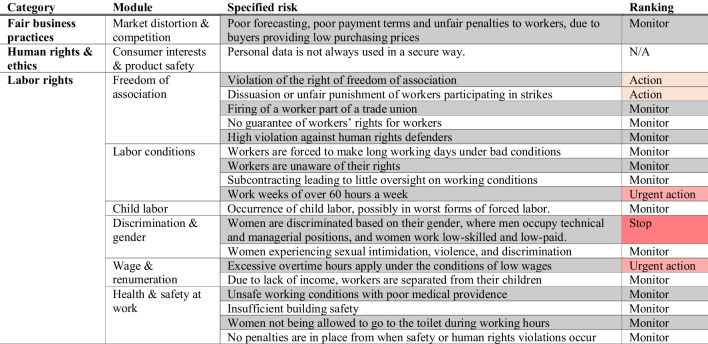
Sector-level risk evaluation results related to the Tier 1 manufacturing organization through the Roadmap to CSR risk management

Insufficient data hindered a complete assessment of the Tier 2 yarn manufacturer for both the Roadmap and IFC Compliance assessment. Also, the CSR Risk Check Tool was unable to generate reports on risks regarding the design or retailing phase, so this assessment and evaluation step was also not conducted for the fashion brand. Consequently, where the IFC Compliance assessment mainly focuses on the fashion brand and Tier 1 T-shirt manufacturer, the Roadmap assessment mainly focuses on the Tier 1 T-shirt manufacturer.

#### Subcategory assessment method

##### Description

**Fig. 4:**
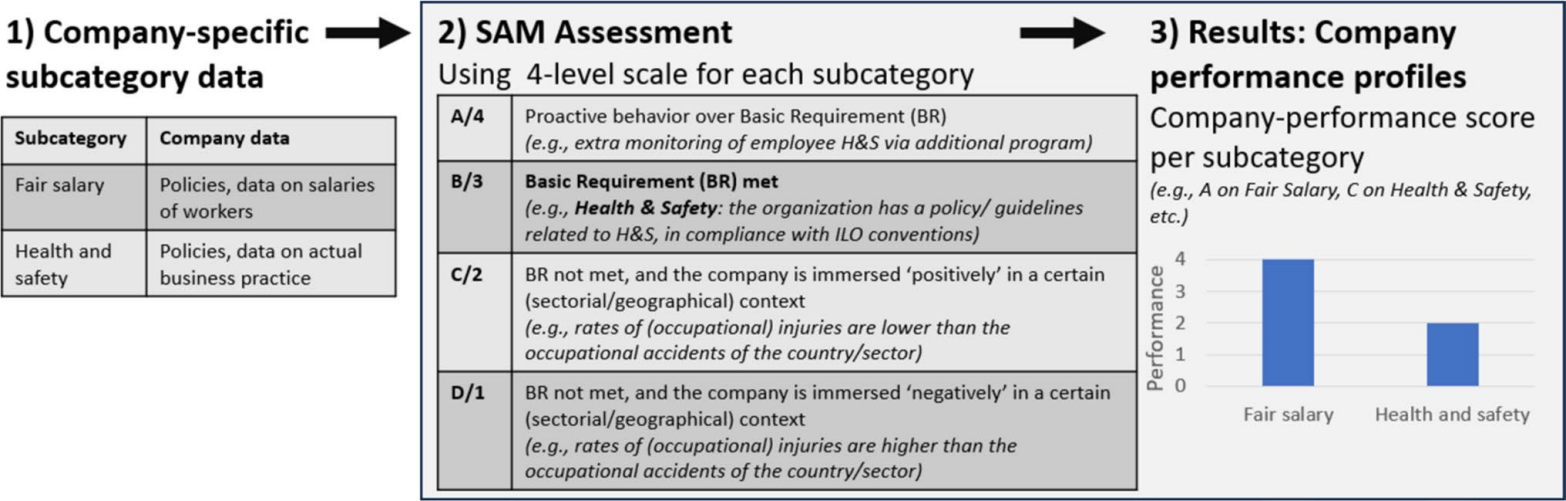
Conceptualization of SAM, visualizing the steps taken to assess data to a formulated four-level reference scale. Own image

Many indicators proposed by UNEP (2020, 2021) are qualitative by nature and relate to company behavior. There is a widely accepted notion of the relevance of organization-oriented assessments — based on the argument of social impacts (e.g., non-discrimination and equal opportunities) being tied to company behavior rather than the function a product delivers (Dreyer et al. 2006; Weidema 2005; Zamagni et al. 2011). This is reflected by the widespread use of qualitative approaches like SAM (Lenzo et al. 2017; Wu et al. 2014). Initially based on the Guidelines, SAM is a RS approach which takes an organizational perspective evaluating social profiles of organizations in a product’s value chain (Ramirez et al. 2014). Its steps (Fig. 4) involve gathering qualitative company-specific data (1), assessing this against 4-level scales per subcategory (2), resulting in company performance profiles per subcategory (3).

As SAM was initially developed as a S-LCIA method for assessing subcategories (Ramirez et al. 2014), it logically necessitates the formulation of a FU during the goal and scope phase. The authors also presented a case study employing SAM, wherein they defined a FU and utilized the worker-hours activity variable to establish cut-off criteria for including organizations in the assessment (Petti et al. 2016). They excluded companies with a share of labor hours below than a certain percentage. However, since the evaluation primarily focuses on company performance and the social context surrounding the product system, impacts are not scaled to the formulated FU.^6^ It is therefore crucial to refrain from labeling SAM as an S-LCIA method, as its orientation remains organization-centric rather than product-oriented.

##### Case study application and illustrative results

SAM requires extensive data collection; therefore, a selection on stakeholder groups (5) and subcategories (16) to assess was made first, guided by a literature review pinpointing key social issues in the garment industry. Surveys adapted from Petti et al. (2016) were set up to gather primary data from the three main organizations. This data was translated to the semi-quantitative SAM framework (Table 6), categorizing organizational performance from A (best) to D (worst) per subcategory, following the scoring framework proposed by Ramirez et al. (2014).^7^

**Table 6:**
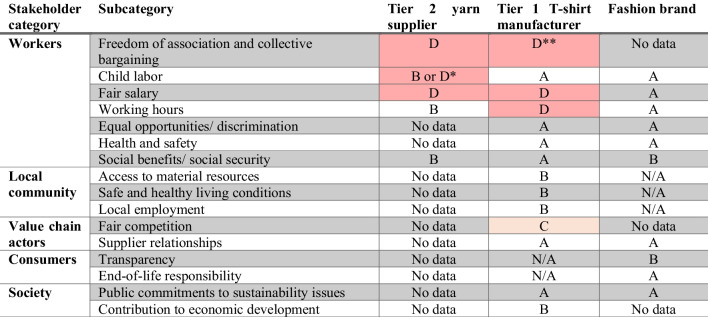
Through SAM-identified organizational performance of the three main companies over 16 prioritized subcategories

A: complying to the Basic Requirement (BR), plus showing proactive behavior by promoting CSR on this aspect amongst suppliers. B: complying to the BR, but not showing any additional proactive behavior. C: not complying to the BR and the organization being immersed in a context described in C (e.g., “a country with low risk on child labor”). D: lowest scoring. Not complying to BR and immersed in a negative context

^*^No information is available if the Tier 2 supplier has a child labor policy in place

^**^Contradicting answers were provided by the Tier 1 manufacturer, leaving this scoring as under high uncertainty levels

## Discussion

Our analysis shows that, because of its product-orientation, S-LCA differs in structure, logic, and scope from other social assessment frameworks, yielding results that vary in nature, depth, and social considerations. Moreover, despite institutional backing and a proposed ISO 14075:2024 standard (International Organization for Standardization 2024), S-LCA cannot yet be considered the social counterpart to E-LCA and LCC due to fundamental differences in computational structure. Nevertheless, the need for comprehensive sustainability assessment of product value chains that include a social dimension remains critical to science and society. Therefore, here, we evaluate our findings from analyzing the landscape of social assessment methods, their methodology, and the compatibility of S-LCA with LCSA frameworks.

### The landscape of social assessment methods

In the current work, we defined S-LCA solely as those methods adopting a quantitative, FU-based LCA structure, in line with the computational structure of LCSA presented in Eq. 1. These were performed as the social database RS method through the two PSILCA activity variable and the PSILCA direct quantification methods. In contrast, we understand the performed IFC Compliance assessment and the Roadmap as social due diligence methods, taking a checklist and scoring approach respectively. Finally, while initially developed as an S-LCIA method (Ramirez et al. 2014), we classified SAM as a scoring method under the RS approach. We refrain from calling SAM an S-LCIA method, as is incompatible with the definition of S-LCA used in this paper: SAM does not adopt a fully quantitative approach and does not allow for life cycle calculations. Table 7 provides an overview of the characteristics of all methods.

**Table 7:** Overview of characteristics of each conducted method

Method	Impact categories	Link to FU/quantification	Impact Assessment description	Life cycle stages covered
Social database: PSILCA — activity variable	Pre-determined set of categories based on the UNEP Guidelines: 69 indicators over 25 subcategories	Via activity variable worker-hours	PSILCA’s Social Impact Weighting method: characterization of risk-assessed inventory flows to the medium risk level. Reference scales as pre-determined in the database	Allows for a full life cycle perspective. However, the database is limited in its level of resolution of sectors, as well as amount of sectors included
Social database: PSILCA — direct quantification	Pre-determined set of categories based on the UNEP Guidelines: 69 indicators over 25 subcategories	Through the direct quantification: the original amount of indicators in relation to the reference flow is taken “directly.”	Normalization as weighted average of raw indicator values. Manually, PSILCA’s reference scales can be used to classify results in risk classes	Allows for a full life cycle perspective. However, the database must be expanded to include more and better fitting sectors so that this is not a cut-off criteria
Checklist: IFC	Pre-determined list of requirements through Performance Standard	N/A	Categorization in no/medium/full compliance	Supply chain focus (stakeholders)
Scoring: Roadmap to CSR Risk Management	Sector-level social risks identified through MVO’s Risk Check online tool	N/A	Risk scoring matrix categorizing risks based on risk likelihood and severity	Supply chain focus (stakeholders)
Scoring: Subcategory Assessment Method (SAM)	Based on UNEP Guidelines	N/A	Evaluating company performance against levels distinguished through formulated requirements	Allows for a life cycle perspective on organizations along a product supply chain

### Methodological evaluation of the five methods

Due to the very local nature of social impacts, data availability poses challenges in S-LCA (Jørgensen et al. 2009). Using generalized unit process data from social databases can act as a screening tool to identify hotspots, reducing the need for primary data (Hauschild et al. 2008). However, current databases are highly aggregated at sector and country level. There will be significant variety within a specific sector in a country, causing this data to be highly uncertain (Jørgensen 2013). This leads to further need for in-depth and specific studies when aiming to thoroughly assess the social impacts of a specific product (Toboso-Chavero et al. 2021). Koese et al. (2022) also note the cost-sensitive aspect of interpreting S-LCA results from social database assessments. Such databases directly scale social risks to product prices, resulting in discrepancies from practitioners using price-data sources (Tragnone et al. 2023), continuously fluctuating material or component market prices, and fundamental methodological issues (Koese et al. 2022). Employing such generic databases may thus compromise the representativeness of an assessment. While customization of default parameters in PSILCA is possible (e.g., defining risk-levels or the number of worker-hours), such manual modifications are both labor-intensive and error-prone due to its monetary- and worker hours–based structure.

The IFC Compliance assessment and the Roadmap are both found to be social due diligence methods, yet they employ different approaches. The Roadmap identifies sectorial risks and links these to a product system, while the IFC Compliance assessment focuses on individual company performance, overlooking sectorial context. While neither perspective can be integrated with E-LCA or LCSA, both qualitative perspectives are deemed important: the Roadmap assessment raises awareness of sector-level risks and facilitates targeted actions to mitigate these risks by correlating severity with the likelihood of occurrence at an organization. Conversely, the IFC Compliance assessment was conducted at policy-level, contributing to enhancing policy-level performance. Together, these — or similar — methods offer a more comprehensive understanding, aiding in reducing specific risks and enhancing policy-level performance. Taking a supply chain perspective, both assessments do not allow for assessing further downstream life cycle stages (e.g., the use stage), which can be covered through S-LCA. SAM does take a life cycle approach in a qualitative way, assessing specific organizations on their social performance, and evaluating the social context surrounding a product system.

All methods reviewed and applied in this paper require substantial (sensitive) data, retrievable from social databases or primary sources like surveys and interviews (Wu et al. 2014), which companies are reluctant to share (Toboso-Chavero et al. 2021). Gathering extensive sensitive primary data poses challenges, relying on stakeholder cooperation (Pollok et al. 2021). Kokubo Roche (2022) highlights issues arising from gathering primary data, on (i) employee reluctance to participate in interviews and surveys, (ii) the time-consuming identification of relevant contacts within a company, (iii) the difficulty accessing data from value chain actors, and (iv) the substantial resource demands across different geographical regions. Therefore, promoting and engaging stakeholder participation is essential. Potentially, narrowing down the system boundary might be needed, which could sacrifice a comprehensive life cycle perspective.

Our evaluation of two S-LCA and three more qualitative social assessment methods (Table 8) highlights their different modeling structures, with results differing considerably in nature, depth, and social topics covered. This leads to significant differences in results between methods, with on its turn might lead to overlooking important social findings when choosing one qualitative method over the other.

**Table 8:** Overview of identified pros and cons of the five methods conducted

Method	Pros	Cons
Social database: PSILCA — activity variable	• Has potential to compatibility with integration in LCSA frameworks • Allows for contribution analysis following E-LCA principles. Hereby, it allows for a thorough assessment of background processes as well, next to only (for feasibility) focusing on main organizations/stakeholders involved through the other methods	• The database includes a limited set of sectors • Inventory- input-modeling of translating physical quantities to monetary values is a highly error-sensitive process • Social flows being worker hours–based makes it difficult correctly reflecting values when adjusting certain flows • Using the worker-hours activity variable leads to incorrectly scaling social aspects to a product system which do not have a causal link with the number of hours of labor performed • Presenting results as “med risk hours” is an ineffective and incomprehensible unit for reflecting product impacts • A very underreported aspect of PSILCA’s characterization factors being derived from a semi-quantitative arbitrary risk scale, making it more of a risk prioritization tool rather than database for quantitative impact assessment • LCA modeling/software experience needed • Highly expensive database license required
Social database: PSILCA — direct quantification	• Has potential to compatibility with integration in LCSA frameworks • Overcomes using the worker-hours activity variable, whereas an LCA structure remains • Identifies social risks at unit process level with their raw values • The social flows reflect their raw value, allowing for easier adjustment and better communication of these values	• The database includes a limited set of sectors • Inventory input modeling of translating physical quantities to monetary values is a highly error-sensitive process • No characterization nor contribution analysis possible, with limited use of (more comprehensible) results • Leaves calculations at inventory level. Manually, LC calculations can be classified in risk classes of the database • LCA modeling/software experience needed • Highly expensive database license required
Checklist: IFC Compliance assessment	• No particular assessment expertise needed • Easy methodology through taking a “checklist” approach • Captures adequate policy-analysis on stated IFC requirements	• The supply chain perspective does not allow for including upstream life cycle stages (e.g., the use stage) • No product-orientation but rather a form of due diligence • Leaves the analysis at policy-level
Scoring: Roadmap to CSR Risk Management	• No particular assessment expertise needed • MVO’s CSR Risk Tool provides exhaustive reports on social risks per sector in a country as background data for the assessment and basis for a hotspot analysis	• The supply chain perspective does not allow for including all life cycle stages (e.g., the use stage) • No product-orientation but rather a form of due diligence
Scoring: Subcategory Assessment Method (SAM)	• No particular assessment expertise needed • Captures stakeholder-perspective of social issues • Integrates qualitative and (semi-) quantitative data • Possibility to assess on all stakeholder and subcategories	• Requires extensive amount of primary sensitive data • Practitioners can formulate reference scales themselves • Time-requiring data collection process

### Compatibility of S-LCA with LCSA frameworks

The diversity of developed S-LCA methods reflects the fragmentation and lack of concrete methods provided by the Guidelines, from which they are built. This results in various interpretations and non-standardized approaches used amongst practitioners (Pollok et al. 2021), e.g., formulating different reference scales or using other criteria for prioritizing stakeholder and/or subcategories. This causes ambiguous and untransparent S-LCA results, solely focusing on negative social impacts (see Di Cesare et al. 2018) or not including all life cycle stages, nor stakeholder categories (Arcese et al. 2017; Gompf et al. 2022; Kokubo Roche 2022). It is argued that S-LCA should encompass both positive (e.g., wealth creation) and negative aspects (e.g., unethically performed processes) (Hauschild et al. 2008), as it is important to also consider social benefits in a product system when assessing socially sustainable production and consumption (Clift 2014). Addressing the discrepancy between the general S-LCA risk-language and positive impacts is in this case crucial. Also, the non-linear behavior of social impacts should be noted: S-LCA targets a context-specific optimal stage of social impacts; whereas E-LCA has a zero-impact goal, this is not always clear in S-LCA. For example, “contribution to economic development” has an ideal % to the country’s GDP, or “fair salary” should reflect at least a living wage.

In its current form, S-LCA does not adequately capture the qualitative nature of social impacts and their causal link to the behavior of value chain actors, making it fundamentally incompatible with LCA and LCC. S-LCA methods do not effectively follow the ISO framework for E-LCA (International Organization for Standardization 2006). Discrepancies in system boundaries result in a lack of uniformity in the A-matrix between S-LCA and E-LCA, and there is a less distinct separation between S-LCI and S-LCIA than in E-LCI and E-LCIA.

On this note, Clift (2014) argues that S-LCA might be more effective when not constrained by an E-LCA-like framework (i.e., adopting a quantitative, FU-based LCA structure). We echo this argument, but still advocate for the further development of S-LCA this way. Where it might be that social assessments are more effective when not constrained by such a framework and qualitative impacts may be lost out of sight, it is the only methods that adopt a quantitative, FU-based structure which meet the criteria for being considered S-LCA and have the potential to be compatible to integration in LCSA frameworks. S-LCA should then be constrained in its scope to capturing correctly scalable indicators only. We see S-LCA taking a role in LCSA on the social dimension, but because of its limited scope of quantifiable and scalable indicators, this role is a less prominent one than E-LCA and LCC can provide on the environmental and economic dimension.

Developing suitable indicators for S-LCA, however, poses a significant challenge. Currently, S-LCA features approximately 70 indicators, making the synthesis of results difficult. We propose condensing the list of used indicators to improve the comparability and communicability of studies that use this method. However, condensing the indicators to a smaller, standardized set raises questions about its exhaustiveness across various contexts. Kokubo Roche (2022) indicates that defining new indicators specific to a study is subjective, requiring expert and stakeholder consultation. It is also considered important to integrate cultural aspects (Pollok et al. 2021) and income inequality (see, e.g., Weidema 2016) when developing indicators. To improve uniformity within the field, we echo Hauschild et al.’s (2008) proposal of using two indicator sets: a mandatory predetermined set representing minimum requirements and a self-determined, more tailored set reflecting culture-, product-, sector-, and company-specifics.

Nevertheless, the potential compatibility with LCSA’s computational structure is the core strength of social database methods. We find that these methods are the most promising for integration into LCSA frameworks as it uses a quantitative, unit process–based approach — albeit at sector level — which allows scaling of social flows to a FU. This scaling, however, presents a major challenge due to the non-quantifiable nature of social aspects, that are not always causally linked to processes in a product value-chain (Wu et al. 2014). Mapping causal links between economic activities with their physical flows (i.e., goods, wastes) and social impacts in a way transferable to a (mathematical) S-LCA model is complex, as many flows stem from higher-level — i.e., country or sector — issues in which an organization operates. Current RS S-LCA approaches that preserve to a certain extent the original E-LCA framing (i.e., using a social database) utilize the activity variable as quantitative relation between value chain organizations and the product.

Although the use of an activity variable brings S-LCA closer to the ISO framework for E-LCA (International Organization for Standardization 2006), its efficacy has been debated. For instance, an issue arises when using the worker-hours activity variable, as the amount of hours only directly applies to certain “worker” indicators, (e.g., occupational accidents, hourly wage, etc.), yet it is used for all stakeholder categories — i.e., local community, value chain actors, consumers, society, and children. For instance, yarn production by the Tier 2 organization is not causally linked to the illiteracy rate in its operating country or sector, leading to incorrect associations between illiteracy and this yarn production.

The recently developed “direct quantification” method (Ciroth and De Bellis 2020) bypasses the use of an activity variable. It offers a more comprehensible assessment using raw inventory values, not reflecting results as “med risk hours.” However, calculations and interpretability are limited as the impact assessment results only reflect a weighted average of the indicator values of the total results. No characterization is included, and no contribution analysis is possible. PSILCA’s arbitrary semi-quantitative risk scale, from which characterization factors are derived (see Appendix I), makes PSILCA more of a risk prioritization tool rather than a comprehensive database for quantitative impact assessment compatible with LCSA frameworks.

The development of other qualitative, organization-oriented approaches is driven by the argumentation of organizational behavior having greater effect on stakeholders than the function a product delivers (Dreyer et al. 2006; Weidema 2005; Zamagni et al. 2011). Assessing product’s social impacts in relation to company behavior adds contextual value that goes beyond the product-centered focus of S-LCA. Thus, fundamentally different (e.g., organization-oriented) methods could complement S-LCA studies to evaluate the broader context of the product without being a part of S-LCA, in the same way that E-LCAs are strengthened when applied with chemical Risk Assessment while remaining two separate methods.

### Limitations of the study

The conclusions raised in this paper are derived from applying methods to an illustrative single case study. Therefore, there are limitations to these findings in terms of granularity of the analysis, the scope and object of the study, and corresponding data availability. Also, we acknowledge that different sectors might use specific methods or indicators to evaluate and prioritize specific social concerns in their value chains and thus deviate from methods as illustrated here. An additional limitation is that IP S-LCA approaches, which also have the potential to be compatible for integration into LCSA, were not evaluated in this study.

## Conclusions and recommendations

We advocate for enhancing FU-based S-LCA, including a limited set of accurately quantifiable social aspects. In the case of RS approaches, such an assessment would take the form of the database method; i.e., using LCA software and a social database for modeling. Currently, only limited takeaways can be drafted from generic results of such S-LCA studies, considering all barriers discussed. To comprehensively assess a product’s social sustainability, S-LCA and more organization-oriented methods should be applied in combination to also evaluate the context surrounding a specific product. S-LCA guidelines would be better served by narrowing its scope exclusively to product-oriented S-LCA methods and augmenting its results through the use of other qualitative social assessment methods. These S-LCA methods should remain restricted to solely including accurately quantifiable indicators, while important context-related aspects should be covered using more suitable, i.e., qualitative and organization-oriented methods. When desired to conduct an LCSA including a thorough social assessment, it is important to take a hybrid approach by integrating different, most fitting, social impact assessment methods complementing the S-LCA. Such a hybrid approach can overcome limitations of existing methods and provide a more comprehensive assessment of social impacts. We emphasize the equal importance of product-oriented S-LCA and organization-oriented social assessment methods.

Ideally, S-LCA should adhere to the ISO framework established for E-LCA and should be compatible with the integration of LCSA. However, this study found that current S-LCA methods do not fully align with this framework. Discrepancies in system boundaries result in a lack of uniformity in the A-matrix between E-LCA and S-LCA, and there is a less distinct separation between S-LCI and S-LCIA. Although the use of an activity variable brings S-LCA closer to the ISO framework for E-LCA, its efficacy has been found deficient.

Currently, the UNEP Guidelines and S-LCA ISO 14075:2024 standard (International Organization for Standardization 2024) are very ambiguous. The E-LCA and LCC community should be further implicated in the development of social dimension and the formulation of S-LCA to ensure improved compatibility in LCSA frameworks. Enhancing S-LCA guidelines is essential for reducing ambiguity on methodology and terminology. Presently, the use of terms like “normalization” do not conform with E-LCA standards. Recognizing the less distinct differentiation between S-LCI and S-LCIA, terms such as “subcategory” or “indicator” are utilized in S-LCI, whereas in E-LCA they would pertain to E-LCIA. It is imperative to clarify and align the terminology with E-LCA standards.

Future research on developing product-S-LCA should be conducted to (i) refining methods to better address non-causally linked social flows, (ii) integrating frameworks to include positive impacts and cultural aspects and addressing the discrepancy between such impacts and S-LCA’s current risk-language, (iii) improving translating physical quantities to monetary values for modeling with S-LCA databases, (iv) evaluating and enhancing characterization models in social databases, and (v) refining social databases on more and higher-resolution sector-level data, increasing its usability and decreasing uncertainty of results. Furthermore, research is required on the compatibility of integration of IP approaches to S-LCA in LCSA frameworks: how its computational structure fits, and its potential to address identified challenges in this paper. It is important to consider compatibility of S-LCA's computational structure over making it a comprehensive method for social assessment, for which on many aspects more qualitative methods are more suitable for.

## Data Availability

The data that support the findings of this study are not openly available due to sensitivity reasons and are available from the corresponding author upon reasonable request.

## Appendix I Modeling details of PSILCA

### Inventory analysis in PSILCA: Foreground modeling

#### Economic flows

As PSILCA is monetary based, physical quantities need to be converted to monetary values. PSILCA’s third version of the database is based on the Eora MRIO database containing data on USD at the rate of 2011 (USD2011) (Maister et al. 2020). The following conversion process was used, following the approach proposed by Koese et al. (2022):

- Version 3.8 of the ecoinvent database was used to translate physical quantities of flows to monetary values. Certain quantities of different components in ecoinvent are assigned a monetary value in Euros at the rate of 2005 (EUR2005) (as found in unit process dataset information in the version 3 database).
- Consequently, a conversion of the ecoinvent prices to EUR2011 (*1.13) was done through using an online inflation tool (Inflation Tool, 2023) and into USD2011 (*1.2952) using the December 31st exchange rate of 2011 (ExchangeRates.org, n.d.), corresponding with the exchange rate used in PSILCA (Maister et al. 2020).

#### Social flows

For the activity variable method, sector-level social flows contain a default value with associated risk level. These risk levels are assigned to indicator values based on labor laws, international conventions, and standards (Maister et al. 2020), but could be modified individually.

The worker-hours variable contains the unit reflecting these social flows. Information should be gathered on how many labor hours were conducted at a certain facility (i.e., unit process) to produce 1 USD of output (i.e., reference flow). This value then should be assigned to all associated social flows.

When the direct quantification method is followed, the units of the social flows consist of their raw value and allow for easy adjustment. For example, for the stakeholder category workers, the indicator for Health and Safety — Accident rate at the workplace is measured in no. of cases per 100,000 employees and year. If you have primary information available on this indicator, that this accident rate contains a number of cases equal to 10, this value can be assigned to this specific social flow.

### Impact assessment in PSILCA

The direct quantification method can be performed through a separate dataset of PSILCA, as described in the PSILCA v3 documentation (Maister et al. 2020).

Using the worker-hour activity variable model in PSILCA, the “Social Impact Weighting Method” is applied, calculating the total impact assessment results through the following equation applied to each unit process (P):

$${C}_{I}=\sum_{F}\sum_{P}{q}_{I,F}\times {b}_{F,P}\times {s}_{P}$$

This equation reflects;

*C*_*I*_: social indicator value for type $I$ (e.g., “children in employment, total”).

*b*_*F,P*_: amount of worker-hours spent per unit of output of the process $P$ on flow $F$

*S*_*P*_: scaling factor for process $P$, calculated by inverting the technology matrix and multiplying by the demand factor–as in E-LCA.

*q*_*I,F*_: characterization factor to express flow contributions in terms of “med risk hours” of flow $F$ to social impact $I$

The social impact weighting method expresses indicator data at medium risk level through using characterization factors (Table 9). Corresponding with each risk level, the characterization factors reflect an arbitrary factor on exponential scale irrespective of a specific indicator. For positive impacts, also opportunity levels are considered, entering the formula in the same way but through less levels (right columns in Table 9). Currently, only one indicator in PSILCA, “Contribution of the sector to economic development,” assesses an opportunity level instead of a risk level (Maister et al. 2020).

**Table 9:** Characterization factors for corresponding risk and opportunity levels for the social impact weighting method in PSILCA

Risk level	Factor	Opportunity level	Factor
Very low risk	0.01		
Low risk	0.1	Low opportunity	0.1
Medium risk	1	Medium opportunity	1
High risk	10	High opportunity	10
Very high risk	100		
No risk / no opportunity	0	No data	0.1

We take the example of a product system consisting of two unit processes, where in process 1 (economic sector 1) 0.26 h of work (i.e., worker-hours), and in process 2 (economic sector 2), 0.18 respective hours are executed, both to produce 1 USD of output.

Following the proposed notation by Heijungs (2022), PSILCA’s $\mathbf{B}$ and $\mathbf{Q}$ matrices of PSILCA on the indicators “children in employment” and “fatal accidents” in this case are as follows:

Note: Children in employment indicator yCE is children employed as a percentage of the total workforce. Fatal accidents indicator yFA is number of fatal accidents per 100,000 employees.

The $$\mathbf{B}$$ matrix here reflects the amount of work hours per economic sector, i.e., unit process, and $$\mathbf{Q}$$ matrix presents the characterization factors. Note that the amount of labor hours for its associated risk-assessed flow per column in $$\mathbf{B}$$ will always be the same within each column, i.e., the number of work hours required to produce 1 USD worth of the product/service from the economic sector: it will take a fixed amount of work hours to produce 1 USA of value irrespective of the social flow. The $$\mathbf{A}$$ matrix in PSILCA is based on the multiregional economic IO database EORA, so sectors are interchanging $$ rather than technologies interchanging products/services like in ecoinvent for E-LCA.

Social flows are assigned one of six risk levels per indicator, i.e., it cannot be assigned to high-risk and low-risk children in employment simultaneously. In building the inventory of social flows, the right risk level for each flow type is picked by assigning a value to the indicator (which is defaulted through data from the database), assigned the fixed work hours required by the sector to produce 1 USD, which omits the other risk levels (i.e., set them = 0) for that flow type.

## Footnotes

1 SSbD is a premarket approach, aiming to integrate safety and sustainability considerations early in innovation processes and throughout the entire lifecycle of a chemical, material, and product (Caldeira et al. 2022; EC 2022). This is done through a stepwise approach, conducting a risk assessment, environmental LCA, and optionally a socio-economic assessment including an S-LCA. At the basis of the SSbD concept lies the European Union (EU) Chemicals Strategy for Sustainability (CSS) (EC 2020), which is part of the EU’s drive towards zero pollution under the European Green Deal.

2 In S-LCA modeling, one unit process can then be seen as a company with an associated (production) process at a specific location, which was the way modeled in our case. If a specific company operates over several locations, these should be modeled as several unit processes — each with their specific social contexts.

3 The total percentual weight of the T-shirt consists of cotton from the Tier 2 yarn supplier, with a 93.5% share. Over 30 chemicals from 15 other suppliers are added, mostly process chemicals which mostly contribute to a 0.01–0.02% of total product weight. These are modeled in the S-LCA, but these suppliers are not contacted for primary data. Keeping in mind the illustrative purpose of the case led us to prioritizing the methodological evaluation of the five different methods.

4 The use of the term “indicator” in inventory is not aligned with how it is used in the context of E-LCA, where this term is used in LCIA only.

5 Normalization in E-LCA terms is defined through ISO 14044 as expressing indicator results relative to well-defined reference information. Here, it implies dividing total impacts by the scaled diagonal of the A matrix. It is important to realize that how the term is used in the context of S-LCA does not refer to normalization in E-LCA. It should be used more precautionary to not cause terminological misunderstandings between S-LCA and E-LCA.

6 Performance results are organization-oriented, meaning these do not differ for 1 kg of product, 100 items of the product, or any other similar product situated in the same social context, i.e., produced by the same company. Ramirez et al. (2014) and Petti et al. (2016) employ the FU and activity variable concepts related to S-LCA, but they apply these concepts differently from S-LCA principles.

7 Here, we refer to the Electronic Supplementary Material 1 and 2 of Ramirez et al. (2014).

## References

[CR1] Arcese G, Lucchetti MC, Massa I (2017) Modeling social life cycle assessment framework for the Italian wine sector. J Clean Prod 140:1027–1036. 10.1016/j.jclepro.2016.06.137

[CR2] Bennema M, Norris G, Norris CB (2022) The social hotspot database V5. NewEarthB. Retrieved from http://www.socialhotspot.org/

[CR3] Caldeira C, Farcal L, Garmendia Aguirre I, Mancini L, Tosches D, Amelio A, Rasmussen K, Rauscher H, Riego Sintes J, Sala S (2022) Safe and sustainable by design chemicals and materials: framework for the definition of criteria and evaluation procedure for chemicals and materials (978–92–76–53264–4). Retrieved from https://publications.jrc.ec.europa.eu/repository/handle/JRC128591

[CR4] Chhipi-Shrestha GK, Hewage K, Sadiq R (2015) ‘Socializing’ sustainability: a critical review on current development status of social life cycle impact assessment method. Clean Technol Environ Policy 17(3):579–596. 10.1007/s10098-014-0841-5

[CR5] Ciroth A, De Bellis A (2020) Direct quantification of indicators in social LCA (beyond worker hours). https://www.youtube.com/watch?v=aY84nRmbZOo&ab_channel=openLCA Accessed 9 Dec 2023

[CR6] Clift R (2014) Social life cycle assessment: what are we trying to do? In: Proceedings of the 4th International Seminar in Social LCA, Social LCA in Progress, Montpellier, pp 19–21

[CR7] Di Cesare S, Silveri F, Sala S, Petti L (2018) Positive impacts in social life cycle assessment: state of the art and the way forward. Int J Life Cycle Assess 23(3):406–421. 10.1007/s11367-016-1169-7

[CR8] Dreyer L, Hauschild M, Schierbeck J (2006) A framework for social life cycle impact assessment. Int J Life Cycle Assess 11:88–97. 10.1065/lca2005.08.223

[CR9] EC (2020) Chemicals strategy for sustainability towards a toxic-free environment. Retrieved from https://circabc.europa.eu/ui/group/8ee3c69a-bccb-4f22-89ca-277e35de7c63/library/dd074f3d-0cc9-4df2-b056-dabcacfc99b6/details?download=true

[CR10] EC (2022) Commission recommendation of 8.12.2022 establishing a European assessment framework for ‘safe and sustainable by design’ chemicals and materials. Retrieved from https://eur-lex.europa.eu/legal-content/EN/TXT/?uri=CELEX:32022H2510

[CR11] Goedkoop MJ, de Beer I, Harmens R, Saling P, Morris D, Florea A, Hettinger AL, Indrane D, Visser D, Morão A, Musoke-Flores E, Alvarado C, Rawat I, Schenker U, Head M, Collotta M, Andro T, Viot J, Wathelet A (2020) Product social impact assessment handbook - 2020, Amersfoort, November 1st, 2020

[CR12] Gompf K, Traverso M, Hetterich J (2022) Applying social life cycle assessment to evaluate the use phase of mobility services: a case study in Berlin. Int J Life Cycle Assess 27(4):603–622. 10.1007/s11367-022-02051-y 35502215 10.1007/s11367-022-02051-y PMC9046070

[CR13] Guinée J (2016) Life Cycle Sustainability assessment: what is it and what are its challenges? In: Clift R, Druckman A (eds) Taking Stock of Industrial Ecology. Springer Cham, pp 45–68. 10.1007/978-3-319-20571-7_3

[CR14] Guinée JB, Heijungs R, Huppes G, Zamagni A, Masoni P, Buonamici R, Ekvall T, Rydberg T (2011) Life cycle assessment: past, present, and future. Int J Environ Sci Technol 45(1):90–96. 10.1021/es101316v 10.1021/es101316v 20812726

[CR15] Harmens R, Goedkoop M (2021) ORIENTING D1.2 Critical evaluation of social approaches. 10.13140/RG.2.2.16478.31042

[CR16] Hauschild MZ, Dreyer LC, Jørgensen A (2008) Assessing social impacts in a life cycle perspective—lessons learned. CIRP Ann 57(1):21–24. 10.1016/j.cirp.2008.03.002

[CR17] Heijungs R (2010) Ecodesign — carbon footprint — life cycle assessment — life cycle sustainability analysis. A Flexible Framework for a Continuum of Tools. Environ Climate Technol 4(1):42–46. 10.2478/v10145-010-0016-5

[CR18] Heijungs R (2022) The revised mathematics of life cycle sustainability assessment. J Clean Prod 350:131380. 10.1016/j.jclepro.2022.131380

[CR19] Heijungs R, Settanni E, Guinée J (2012) Toward a computational structure for life cycle sustainability analysis: unifying LCA and LCC. Int J Life Cycle Assess 18(9):1722–1733. 10.1007/s11367-012-0461-4

[CR20] Hunkeler D (2006) Societal LCA methodology and case study (12 pp). Int J Life Cycle Assess 11(6):371–382. 10.1065/lca2006.08.261

[CR21] Hunkeler DJ, Lichtenvort K, Rebitzer G (2008) Environmental life cycle costing. SETAC. 10.1201/9781420054736

[CR22] IFC (2012) Performance standards on environmental and social sustainability. Retrieved from https://www.ifc.org/en/insights-reports/2012/ifc-performance-standards

[CR23] International Organization for Standardization (2006) Environmental management life cycle assessment — principles and framework. (ISO Standard No. 14040:2006). Retrieved from https://www.iso.org/standard/37456.html

[CR24] International Organization for Standardization (2024). Environmental management — principles and framework for social life cycle assessment. (ISO Standard No. 14075:2024). Retrieved from https://www.iso.org/standard/61118.html

[CR25] Jørgensen A (2013) Social LCA—a way ahead? Int J Life Cycle Assess 18(2):296–299. 10.1007/s11367-012-0517-5

[CR26] Jørgensen A, Hauschild MZ, Jørgensen MS, Wangel A (2009) Relevance and feasibility of social life cycle assessment from a company perspective. Int J Life Cycle Assess 14(3):204–214. 10.1007/s11367-009-0073-9

[CR27] Klöpffer W (2008) Life cycle sustainability assessment of products. Int J Life Cycle Assess 13(2):89–95. 10.1065/lca2008.02.376

[CR28] Koese M, Blanco CF, Vert VB, Vijver MG (2022) A social life cycle assessment of vanadium redox flow and lithium-ion batteries for energy storage. J Ind Ecol 27(1):223–237. 10.1111/jiec.13347

[CR29] Kokubo Roche A (2022) Assessing the social performance and social risks of wastewater treatment systems through social life cycle assessment Master thesis, Leiden University. https://repository.tudelft.nl/islandora/object/uuid%3A6a9e47a6-6f13-4538-b904-407a2ce24332. Accessed 03-09-2023

[CR30] Kühnen M, Hahn R (2017) Indicators in social life cycle assessment: a review of frameworks, theories, and empirical experience. J Ind Ecol 21(6):1547–1565. 10.1111/jiec.12663

[CR31] Lehtonen M (2004) The environmental–social interface of sustainable development: capabilities, social capital, institutions. Ecol Econ 49(2):199–214. 10.1016/j.ecolecon.2004.03.019

[CR32] Lenzo P, Traverso M, Salomone R, Ioppolo G (2017) Social life cycle assessment in the textile sector: an Italian case study. Sustainability 9(11):2092. 10.3390/su9112092

[CR33] Maister K, Di Noi C, Ciroth A, Srocka M (2020) PSILCA database v.3 documentation. Version 1. Retrieved from https://psilca.net/wp-content/uploads/2020/06/PSILCA_documentation_v3.pdf

[CR34] Mazzi A (2020) Chapter 1 – introduction. Life cycle thinking. In: Ren J, Toniolo S (eds) Life Cycle Sustainability Assessment for Decision-Making. Elsevier, pp 1–19. 10.1016/B978-0-12-818355-7.00001-4

[CR35] Mesa Alvarez C, Ligthart T (2021) A social panorama within the life cycle thinking and the circular economy: a literature review. Int J Life Cycle Assess 26(11):2278–2291. 10.1007/s11367-021-01979-x

[CR36] MVO (2020) Getting started with due diligence. https://www.mvorisicochecker.nl/en/roadmap-csr-risk-management. Accessed 07-10-2023

[CR37] Norris GA (2006) Social impacts in product life cycles - towards life cycle attribute assessment. Int J Life Cycle Assess 11(S1):97–104. 10.1065/lca2006.04.017

[CR38] Petti L, Sanchez Ramirez PK, Traverso M, Ugaya CML (2016) An Italian tomato “Cuore di Bue” case study: challenges and benefits using subcategory assessment method for social life cycle assessment. Int J Life Cycle Assess 23(3):569–580. 10.1007/s11367-016-1175-9

[CR39] Pollok L, Spierling S, Endres H-J, Grote U (2021) Social life cycle assessments: a review on past development, advances and methodological challenges. Sustainability 13(18):10286. 10.3390/su131810286

[CR40] Ramirez PKS, Petti L, Haberland NT, Ugaya CML (2014) Subcategory assessment method for social life cycle assessment. Part 1: methodological framework. Int J Life Cycle Assess 19(8):1515–1523. 10.1007/s11367-014-0761-y

[CR41] Ramos Huarachi DA, Piekarski CM, Puglieri FN, de Francisco AC (2020) Past and future of social life cycle assessment: historical evolution and research trends. J Clean Prod 264:121506. 10.1016/j.jclepro.2020.121506

[CR42] Toboso-Chavero S, Madrid-López C, Villalba G, Gabarrell Durany X, Hückstädt AB, Finkbeiner M, Lehmann A (2021) Environmental and social life cycle assessment of growing media for urban rooftop farming. Int J Life Cycle Assess 26(10):2085–2102. 10.1007/s11367-021-01971-5

[CR43] Tragnone BM, Serreli M, Arzoumanidis I, Pelino CA, Petti L (2023) Using the Product Social Impact Life Cycle Assessment (PSILCA) database for product comparison: Confetti case study. Int J Life Cycle Assess 28(8):1031–1053. 10.1007/s11367-023-02173-x 10.1007/s11367-023-02173-x PMC10153778 37363086

[CR44] UNEP (2020) Guidelines for social life cycle assessment of products and organizations 2020. Retrieved rom Retrieved from http://www.unep.fr/shared/publications/pdf/DTIx1164xPA-guidelines_sLCA.pdf

[CR45] UNEP (2021) Methodological sheets for subcategories in social life cycle assessment (S-LCA) 2021. Retrieved from https://www.lifecycleinitiative.org/wp-content/uploads/2021/12/Methodological-Sheets_2021_final.pdf

[CR46] Valdivia S, Backes JG, Traverso M, Sonnemann G, Cucurachi S, Guinée JB, Schaubroeck T, Finkbeiner M, Leroy-Parmentier N, Ugaya C, Peña C, Zamagni A, Inaba A, Amaral M, Berger M, Dvarioniene J, Vakhitova T, Benoit-Norris C, Prox M., . . . Goedkoop M (2021) Principles for the application of life cycle sustainability assessment. Int J Life Cycle Assess 26(9):1900–1905. 10.1007/s11367-021-01958-2

[CR47] van Dulmen N (2023) Weaving ‘social’ into LCA: a methodological comparison of social life cycle assessment with purely qualitative social sustainability assessment Master thesis, Leiden University. https://repository.tudelft.nl/islandora/object/uuid%3A19f854fc-50b7-46f2-9892-96a06af3051c?collection=education

[CR48] Weidema BP (2005) ISO 14044 also applies to social LCA. Int J Life Cycle Assess 10(6):381–381. 10.1065/lca2005.11.002

[CR49] Weidema BP (2016) The social footprint—a practical approach to comprehensive and consistent social LCA. Int J Life Cycle Assess 23(3):700–709. 10.1007/s11367-016-1172-z

[CR50] Wu R, Yang D, Chen J (2014) Social life cycle assessment revisited. Sustainability 6(7):4200–4226. 10.3390/su6074200

[CR51] Zamagni A, Amerighi O, Buttol P (2011) Strengths or bias in social LCA? Int J Life Cycle Assess 16(7):596–598. 10.1007/s11367-011-0309-3

